# Interactive association of maternal education and peer relationship with oppositional defiant disorder: an observational study

**DOI:** 10.1186/s12888-021-03157-7

**Published:** 2021-03-22

**Authors:** Ming-Chia Liu, Jung Chen Chang, Chau-Shoun Lee

**Affiliations:** 1 Department of Psychiatry, MacKay Memorial Hospital, Taipei, Taiwan; 2 School of Nursing, College of Medicine, National Taiwan University, Taipei, Taiwan; 3 Department of Nursing, National Taiwan University Hospital, Taipei, Taiwan; 4 Department of Medicine, MacKay Medical College, New Taipei City, Taiwan

**Keywords:** Oppositional defiant disorder, Mother-child relations, Peer interaction, Illicit substance use

## Abstract

**Background:**

The objectives of this research were to gain insights on the interactive effects, by measuring familial and peer-related risk factors in youths with oppositional defiant disorder (ODD).

**Methods:**

Participants were college students recruited nationwide, with age between 18 and 25. Through the consensus of expert meetings, a set of questionnaires were used to evaluate the familial status, participant’s peer group conditions, high-risk environment of illicit substance use, and oppositional symptoms. The logistic regression was performed to see the independent and interactive risk factors for ODD.

**Results:**

A total of 981 subjects were enrolled. Six variables significantly associated with ODD at the multivariate logistic regression, including male, night division, poor academic performance, high risk environment, peer with illicit substance use and high maternal education level. High maternal education exerted independent protective effect on the development of ODD (adjusted odds ratio, aOR = 0.65, 95% CI = 0.44–0.99). Peer with illicit substance use was more likely to associate with ODD in the low maternal education group. The 2-way interactive effect of maternal education and peer with substance use on the development of ODD was OR = 4.96 (2.96, 8.31).

**Conclusion:**

The present study highlights the influence of maternal education level to ODD and its interaction with peer of illicit substance use. Our findings imply that the familial attachment and peer interaction are essential stages for the development of human behavior.

**Trial registration:**

The research protocol was reviewed and approved by the ethical review committee of National Taiwan University Hospital (number 201505057RINC) and registered at clinical trial systems at National Taiwan University. In addition, subjects’ information was anonymous and de-identified prior to any analysis.

**Supplementary Information:**

The online version contains supplementary material available at 10.1186/s12888-021-03157-7.

## Background

About one in five children and youths met criteria for a mental disorder but less than one-third had received a mental health professional service in a Canadian study [1]. To strengthen prevention and early intervention efforts by depicting familial and peer influences are essential to meet the mental health needs of youths. Of the mental disorders in youths, oppositional defiant disorder (ODD) represents enduring patterns of argumentative, disobedient attitude toward authority figures, characterized with irritable mood and vindictiveness. ODD is one of the most commonly encountered clinical disorders in children and adolescents. The clinician is most often alerted when problems with oppositionality, vindictiveness, negativistic and hostile behavior which create a significant disturbance in social, academic, or occupational functioning [2]. Data from Avon Longitudinal Study of Parents and Children have shown individuals with ODD were likely to develop anxiety, depression or conduct disorder in the long term follow-up [3]. The Cambridge study on delinquency also found that ODD persons had higher rate of hospitalization or being registered disabled in their middle age [4]. As ODD is among the most common reasons referred to mental health professionals, primary care physicians are positioned to assess youth at risk of ODD and refer their families to community programs [5].

Both familial and peer factors had been found to associate with the ODD. Familial factors, such as parent emotional regulation and cohesion, are related to child ODD symptoms and understanding child behavioral problems within the family context is important [6]. Harsh inconsistent discipline, low warmth and involvement, and high criticism parenting styles were considered risky for the ODD [7]. The parenting style might correlate with children’s externalizing behaviors [8]. Parents’ own antisocial behavior also played a role on the development of children’s oppositional problems [9]. During childhood and adolescence, the oppositional child may be rejected by non-deviant peers and tends to attract other deviant ones. The affiliation with deviant peers were more likely to reinforce mutual antisocial behaviors [10]. The poor social skills and lack of prosocial behaviors were common among children with ODD [11]. *These familial and peer factors were found to influence children’s defiance through parenting practices and social skill learning at peer interactions. Also, social learning theories have shown that individual who witnesses more deviant behaviors from their parents or peers would model the advantage of these behaviors, thus promoting aggression and oppositional behaviors* [12]*.*

Although various familial and peer factors were explored for their impact on ODD, the interactive effects were seldom examined. In the present study, we planned to figure out the independent and interactive relationship of these 2 types of risk factors with the ODD. *Our hypothesis was that high educated parents may be a protective factor toward the development of ODD. And deviant peer group, such as those with substance use or going to risky environment, may increase the risk of developing ODD.* The sample was coming from a national project of Screening for Illicit Substance Use in College (SISUC).

## Methods

### Study design

This study used data from a campus prevention program, the SISUC, which involved about 80% of 151 colleges and universities nationwide [13]. At the first stage, we sent an invitation letter to school personnel in the office of Student Affairs and consecutively recruited schools into the SISUC study. A cross-sectional survey with non-randomized sampling in 14 universities were recruited to join the early phase of the SISUC study. The above 14 universities located in different regions of Taiwan and were public or private supported, and either general- or vocational-oriented. A set of self-report questionnaires were administered to identify the high-risk population and risk factors for illicit substance use. (supplement 1) The measures were given to classes of students and the participants, who understood the purpose and value of the research, might hand in completed questionnaires under anonymity. The main reasons of non-participation were time-limit or not interested and the averaged acceptance rate was 75% among invited students.

### Study participants

Study participants were college or university students recruited across northern, central and southern Taiwan in 2014, with age between 18 and 25. We contacted school nurses, psychologists, teachers, mentors, or counselors and invited the students to participate in the program through those staff once they meet the inclusion and exclusion criteria. *The inclusion criteria included:1) being Taiwanese citizen, 2) aged 18 to 25, 3) currently studying in day or night division of the college or university, 4) with no diagnosed developmental delay that may compromise their understanding of study purpose. The exclusion criteria include:1) participants with severe mental illnesses, 2) physical disabilities, or 3) cognitive impairment.* Each participant was given a written instruction to inform the study related information before participation. The researchers or research assistants explained the purpose and procedure of the program to the participants in classrooms and collected the questionnaire directly under their consents. *The original estimated size of sample was at least 906 based on logistic regression with odds ratio 1.45, alpha error probability 0.05, having 80% power, and 10–20% attrition rate.* The response rate was 75%. They were told that the data of questionnaires would be kept confidential and have no impact to their academic evaluation.

### Study measures

#### Demographic characteristics

The demographic information collected was age, sex, day or night division of school, academic performance, personal income, family type, family income, and parental working status and educational level. Poor academic performance meant failure in more than half of their curricula in a semester. Personal income was divided into three levels: no income, moderate income (below 12,000 Taiwan dollars (TWD) per month) and high income (over 12,000 TWD per month). The single-parent and two-parent family types were also reported. Family economic status was categorized as affluence, balanced, and liabilities. Parental working status were analyzed separately as unemployed or employed father and unemployed or employed mother. Paternal and maternal educational levels were recorded as low and high levels (≦9 years and > 9 years).

### The youth risk behavior surveillance system (YRBSS)

The open website YRBSS was modified and used to survey 6 categories of health or risk behaviors in youth and young adults, including behaviors that contribute to unintentional injuries and violence. This system updates every 2 years with satisfactory reliability [14, 15].

### The semi-structured assessment for drug dependence and alcoholism (SSADDA)

SSADDA was designed according to DSM-IV [16] for assessment of drug and alcohol abuse. It also evaluated the individual’s oppositional behaviors and relevant environmental problems. SSADDA had good inter-rater reliability [17].

### The Chinese version of the schedule for affective disorders and schizophrenia for school-age children-epidemiologic version (K–SADS–E)

K–SADS–E was a semi-structure diagnostic interview scale, developed for assessing youth mental disorders [18]. It has been translated into several languages and used worldwide. The Chinese version has been tested and showed good inter-rater reliability and convergent and divergent validity with other corresponding clinical questionnaires [19].

The relevant items from above measures were integrated into a set of questionnaires through the consensus of expert meetings. This set of questionnaires was used to evaluate the participant’s peer group conditions, personal history, and high-risk environment of illicit substance use, and to screen behavior problems such as oppositional and conduct disorders.

### Assessment of oppositional disorder

We selected 8 questions out of Chinese K-SADS-E according to DSM-5 criteria to detect ODD [20]. The items included “loses temper”, “argue with adult/authority figures”, “disobey rules or requests”, “deliberately annoying others”, “blame others for own mistakes”, “touchy and easily annoyed”, “resentful”, and “vindictiveness”. The participant meeting more than 4 criteria was considered as a possible case.

### Statistical analyses

Statistical analysis was conducted using R v3.5.0 (R core team, 2014). The univariate regression analysis was performed to find the correlation between each risk factor, such as demographic or clinical characteristics, and ODD. The multivariate regression was then carried out to estimate the independent and interactive effects of co-variates on the ODD. *The potential confounders in this study included demographic backgrounds (*i.e.*, age, sex, economic status, family type, parental employment), schooling factors (*i.e.*, day or night division, academic performance), peer influences (peer with conduct behaviors), and environmental factors, such as high risk environment for illicit substance abuse. All those potential confounders were considered in the statistical analyses. We performed a post-hoc analysis by stratification of maternal education level.* The *crude* odd ratio (OR), adjusted OR and 95% confidence interval were calculated and the *p* < 0.05 was considered as the significant level. *There were few missing data (< 1%) for each variable because of the nature of voluntary participation with careful preparation and clear explanation beforehand as well as friendly reminding to check for blanks by research assistants during the process. We used missing value imputation to address missing data for statistics, such as using the same school averaged income level for missing income. A questionnaire could be valid only if the missing items were less than 20% of the entire questionnaire booklet.* As we intended to understand the association of familial attachment and peer interaction with the case of ODD, a post-hoc analyses were performed on the relevant variables.

## Results

A total of 981 subjects between 18 and 25 years of age were enrolled from 14 schools. Social demographic characteristics associated with ODD are shown in Table 1. In the univariate analyses, male (OR = 2.43), study in night division providing classes in the evening (OR = 2.12), living in a single-parent family (OR = 1.75), poor academic performance (OR = 1.95), high personal income (OR = 2.25), going to high risk environment of illicit substance use (OR = 5.61), and peer with conduct behavior (OR = 1.77,) or illicit substance use (OR = 2.78) were significantly correlated with the possible case of ODD. Comparing to the low educational level, higher maternal education was less likely to develop ODD (OR = 0.64).

**Table 1:** Sociodemographic data associated with oppositional defiant disorder in the univariate and multivariate logistic regressions (*n* = 981)

Variables	n (%)	Crude OR (95% CI)	*p*-Value	Adjusted OR (95% CI)	p-Value
Age Range (y)	18–25				
Sex					
Female	534 (54.43)	1			
Male	**447 (45.56)**	**2.43 (1.72, 3.45)**	< 0.001	**1.94 (1.34, 2.83)**	0.001
Division Of College					
Day Division	488 (49.74)	1			
Night Division^a^	**493 (50.25)**	**2.12 (1.50, 3.02)**	**< 0.001**	**1.74 (1.19, 2.56)**	**0.005**
Family Type			0.002		
Double-Parent	256 (26.09)	1			
Single-Parent	725 (73.91)	1.75 (1.22, 2.49)			
Father Working Status			0.29		
Unemployed	65 (6.63)	1			
Employed	916 (93.37)	1.39 (0.73, 2.5)			
Father Education Level			0.74		
Low Education	266 (27.11)	1			
High Education	715 (72.88)	0.94 (0.65, 1.37)			
Mother Working Status			0.567		
Unemployed	336 (34.25)	1			
Employed	645 (65.74)	0.90 (0.63, 1.28)			
Mother Education Level			0.016		0.042
Low Education^d^	229 (23.34)	1		1	
High Education	752 (76.66)	0.64 (0.44, 0.93)		0.65 (0.44, 0.99)	
Poor Academic Performance^c^	138 (14.06)	1.95 (1.27, 2.96)	0.002	1.86 (1.15, 2.98)	0.011
Peer with Conduct Behavior	397 (40.46)	1.77 (1.26, 2.48)	0.001		
Peer With Illicit Substance Use	374 (38.12)	2.78 (1.98, 3.92)	< 0.001	2.11 (1.45, 3.07)	0.0001
High Risk Environment of Illicit Substance Use	410 (41.79)	5.61 (3.86, 8.29)	< 0.001	5.64 (3.83, 8.45)	< 0.0001
Personal Income^b^					
No Income	309 (31.49)	1	< 0.001		
Moderate Income	252 (25.68)	1.06 (0.66, 1.67)	0.813		
High Income	420 (42.81)	2.25 (1.54, 3.32)	< 0.001		
Family economic status					
Affluence	264 (26.91)	1	< 0.001		
Balanced	529 (53.92)	0.62 (0.37, 1.04)	0.717		
Liabilities	188 (19.16)	0.92 (0.61, 1.43)	0.071		

*OR* odds ratio; *CI* confidence interval; crude OR for the univariate regression and adjusted OR for the multivariate regression

^a^ A division providing evening classes for those working during the daytime

^b^Moderate income, below 12,000 Taiwan dollars (about 400 USD) per month; high income, over 12,000 Taiwan dollars per month

^c^Poor academic performance meant failure in more than half of their curricula in a semester

^d^Educational level: low≦9 years and high> 9 years

Numbers of missing data for each variable were as below: 85 for age, 2 for sex, 5 for division, peer with conduct behavior, peer using illicit substance, and high risk environment, 6 for family type, 68 for father’s work, 93 for father’s education, 28 for mother’s work, 76 for mother’s education, 13 for poor academic performance, 77 for personal income, and 63 for family economic status

Table 1*also* shows the adjusted odds ratio of demographic, familial and peer factors on the risk of ODD. Six variables remained significant at the multivariate logistic regression, including male, night division, poor academic performance, high risk environment, peer with illicit substance use and high maternal education level. High maternal education persisted to exert independent protective effect on the development of ODD (aOR = 0.65).

At a further inspection, we stratified the familial factor, maternal educational level, to analyze the differential impact of peer factors on the ODD. In the Table 2, two factors exerted distinct impact on the presence of ODD. Peer with illicit substance use was more likely to associate with ODD in the low maternal education group, but study in the night division made the significant effect in the high level counterpart. We in particular calculated the 2-way interactive effect of maternal education and peer with illicit substance use on the development of ODD, and O.R. = 4.96 (2.96, 8.31) was found.

**Table 2:** Adjusted odds ratio of demographic and peer risk factors for the ODD stratified by maternal education level

	High Maternal Education		Low Maternal Education	
	aOR (95% CI)	p	aOR (95% CI)	p
High-Risk Environment*	5.55(3.20, 10.01)	< 0.001	5.11 (2.45, 11.28)	< 0.001
Peer with illicit Substance Use	1.72 (1.01, 2.93)	0.045	2.82 (1.35, 6.00)	0.01
Male	1.75 (1.03, 2.99)	0.039	2.96 (1.42, 6.41)	0.01
Poor Academic Performance	1.78 (0.89, 3.47)	0.096	1.87 (0.72, 4.73)	0.19
Night Division	2.67 (1.55, 4.71)	< 0.001	0.95 (0.46, 1.99)	0.90

Note: The 2-way interaction, mother with low education level x peer with illicit substance use, was calculated: Odds Ratio (95% CI) = 4.96 (2.96, 8.31), p-value < 0.0001

aOR = adjusted odds ratio;

*high-risk environment meant high risk environment of illicit substance abuse

## Discussion

Our study investigated the associated risk factors for ODD and 6 factors were found to exert independent association, including gender, campus problems, peer interaction and familial variables. We specifically inspected the interactive impact of peer and familial factors on the presence of ODD. The high maternal education level provided a significant protective effect on the male college student at facing the substance enabler in peers.

The current study showed young adult male (age 18–25) was more likely to have ODD than female, which is consistent with previous epidemiology surveys. The gender is constantly a significant predictor for oppositional and further antisocial behavior [21]. However, the prevalence rate of ODD in boys outnumber girls before adolescent, the male predominance existing but declining among adolescent and adult samples. This sex difference of ODD is possibly affected by sociocultural factors, apparent in Western society but not so significant for non-Western culture [22].

As to the influencing factors in family, it is suggested that highly educated mother may use the more sensitive parenting style instead of controlling way [23, 24]. These mothers are likely to know about the importance of keeping consistent and warm parenting style, which thus reduces the externalizing problems in youths [7, 25]. Moreover, the better maternal working memory or cognitive control of emotion along with decreased harsh reactive parenting was found to lessen the oppositional reactivity at interaction with children [26]. Our finding that mother with higher educational level was a protective factor for the development of ODD is compatible with those findings.

In contrast, low educated mothers were more neglectful and this parenting style itself increased the risk of developing ODD in children [27]. Our survey showed that the concurrence of low maternal education level and peer with illicit substance use exerted the highest risk for having the ODD. It is possible that the low educated mother uses dysfunctional parenting style and is less likely to monitor her child’s peer relationship and whereabouts. As the child, particularly boy, had delinquent peers around, he was at risk to increase delinquent behavior and developing oppositional behavior. The illicit substance use in adolescents may predict future conduct problems [28]. Youths with deviant peers would increase their rates of antisocial behaviors after joining a gang, which risk reduces if leaving the gang [29]. The maternal support had not only direct effect on reducing the delinquency but also acting indirectly on deviant peer affiliation [30].

The exposure to high risk environment of illicit substance use persisted highly associated with the ODD despite the protective effect of high maternal education level. A student studying in the night division was more likely to have ODD when mother well educated than with a low educated mother. Studying in night division might be the result of low academic achievement at senior high school or during university entrance exam, particularly for students in higher socioeconomic family. The previous study has showed that the low IQ and low school achievement are important predictors of ODD [31]. Students with low performance may encounter unrewarding experiences through the school lives and turn out to seek support from deviant peers. As deviant youths get together, they possibly reinforce mutual delinquent behaviors [10]. The interaction also increases the risk of developing ODD. At another direction, ODD is commonly a disorder of childhood. Its symptoms significantly interfere with social or interpersonal relationships at primary and middle schools. The persistence of impairment associated with ODD into young adulthood may make the patient enter a night division of college despite the socioeconomic advantage. This phenomenon also calls for a reconsideration of ODD as a disorder limited to childhood [32].

Despite those intriguing findings, our survey is limited by its cross-sectional design and temporal relationship among risk factors and ODD cannot be well defined. *Therefore, we could not confirm the causal direction of risk factors and the disorder.* Secondly, the definition of ODD is according to the self –administered questionnaire instead of interview, thus not a formal diagnosis. *Though the questionnaire is made from diagnostic criteria, potential bias might come from the different interpretation of the symptom questions between participants and experts. However, the present assessment of ODD symptoms offered the advantage of variability in a non-clinical sample. Moreover,* the sample was all from college students, not the community population. *The selection bias exists and generalizability is restricted. We made every possible effort to address the potential sources of bias. First, we recruited participants from both general and vocational colleges and universities, from both public and private schools, from urban and rural areas, from day and night divisions, and from more than half counties nationwide. Second, we provide leaflets, invitation letter, and oral explanation to increase the understanding of self-report questionnaire for participants. We also address that all the answers were processed anonymously and aimed for medical or educational concern not for any legal concern or persecution purpose to increase the truthfulness of the self-reports. Third, we handled the data carefully and rechecked with expert penal from time to time to verify the findings comparable to clinical impressions and campus real world conditions. Forth, we used multivariate regression and calculate the receiver operating characteristic curve (ROC curve) to see the sensitivity and specificity of our model. The interaction ROC curve were 0.79, indicating the acceptable discrimination of our model.*

## Conclusions

This study found that family and peer factors interacted to exert the highest effect for developing ODD. It gives implications of importance at developing relevant programs to prevent college students from poor long-term outcome. In addition to enhance supportive parenting skills, positive campus experiences might help the student resisting the risky peer influence [33, 34]. *The concern of the generalizability (external validity) is a construct that attempts to answer whether we can use the results of the current study in college students other than those enrolled in the study. The participants’ profiles, such as demographics and social-economic status (SES), were similar to general college students in Taiwan. However, it needs cautions to penalize the findings to other counties with significantly non-comparable SES or without similar healthcare and educational resources. In order to increase the generalizability, further study may need more rigorous design, such as collect data from the general population, enroll ethnical minorities, conduct an international collaborative study, perform longitudinal* trend analysis to increase the external validity.

## Supplementary Information

**Additional file 1.**

## Data Availability

Data are available upon reasonable request, subject to the required approvals. All data were obtained by individual self-reported questionnaire. Access to the statistical code and the dataset can be requested from the corresponding author at jungchenchang@ntu.edu.tw. Requests will be granted after consideration by the SISUC Research Group, agreement by the National Taiwan University Hospital Research Ethics Committee.

## Abbreviations

ODD: Oppositional defiant disorder
SISUC: Screening for Illicit Substance Use in College
TWD: Taiwan dollars
YRBSS: The Youth Risk Behavior Surveillance System
SSADDA: The Semi-Structured Assessment for Drug Dependence and Alcoholism
K–SADS–E: The Chinese version of the Schedule for Affective Disorders and Schizophrenia for School-Age Children-Epidemiologic Version
OR: Odd ratio
aOR: Adjusted OR
IQ: Intelligence quotient
ROC curve: Receiver operating characteristic curve
SES: Social-economic status
