# Supplementary material for: Interactive association of maternal education and peer relationship with oppositional defiant disorder: an observational study
Source: BMC Psychiatry. 2021 Mar 22;21:160. doi: 10.1186/s12888-021-03157-7 (PMC7983394; doi:10.1186/s12888-021-03157-7)
Supplement: Supplementary file 1 — Additional file 1. [file 12888_2021_3157_MOESM1_ESM.pdf]

Title page

Interactive association of maternal education and peer relationship with oppositional defiant disorder：An observational study

Health screening questionnaire for college students

Ming-Chia Liu, M.D.[1]

Jung Chen Chang, R.N., Ph.D. [2,3]

Chau-Shoun Lee, M.D., Ph.D.[4,5*]

[1] Attending Physician, Department of Psychiatry, MacKay Memorial Hospital, Taipei, Taiwan

[2] Associate Professor, School of Nursing, College of Medicine, National Taiwan University, Taipei, Taiwan

[3] Vice Chair, Department of Nursing, National Taiwan University Hospital, Taipei, Taiwan

[4] Associate Professor and Deputy Chief, Department of Medicine, MacKay Medical College, New Taipei City, Taiwan

[5] Senior Attending Physician, Department of Psychiatry, MacKay Memorial Hospital, Taipei, Taiwan

***Corresponding Authors and Reprint Requested to:**

Chau-Shoun Lee

Department of Medicine, MacKay Medical College, No.46, Sec. 3, Zhongzheng Rd., Sanzhi Dist., New Taipei City, 25245, Taiwan; Department of Psychiatry, MacKay Memorial Hospital, Taipei, Taiwan

E-mail: csleepsyc@gmail.com

Tel 886-972212137

Health screening questionnaire for college students

I. Basic information

1. **Division of College**： □ Day division □ Night division
2. **Sex**：□ Male □ Female
3. **Department**：__________ / **Grade**：__________
4. **Age**：_________
5. **Have you ever failed in 1/2 or 2/3 of your subjects?** □ Yes □ No
6. **What is your parent's marital status?**
   □Married or live together □Separated □Divorced □Never married
   □Widowed(□Father passed /□Mother passed) □Remarried
7. **What are your parents' education levels?**
   Father: □ Elementary school □Junior high school (9th grade) □Senior high school □ College/University □Graduate institute □Not applicable/Unknown
   Mother: □ Elementary school □Junior high school (9th grade) □Senior high school □College/University □Graduate institute □Not applicable/Unknown
8. **Current residency status:** □Live with parents □Dormitory at school
   □Rent a room □Live with relatives □Others_________________
9. **Do you have a part-time job?**
   □No □Yes, less than $6000 TWD/month □Yes, $6001~12000 TWD/month
   □Yes, $12001~20000/month □Yes, more than $20001 TWD/month
10. **What is your parent's current working status?**
    Father: □Household □Unemployed □Industry/architecture/engineering
    □Agriculture/forestry/fishing/animal husbandry □Business(Self-employed)
    □Business(Employed) □Military/Police □Education/Government employee
    □Medicine/Health □Hospitality □Transportation □Others
    Mother: Household □Unemployed □Industry/architecture/engineering
    □Agriculture/forestry/fishing/animal husbandry □Business(Self-employed)
    □Business(Employed) □Military/Police □Education/Government employee
    □Medicine/Health □Hospitality □Transportation □Others
11. **How is your familial economic condition?**
    □Liability, difficult to afford the daily expense
    □Sometimes difficult to afford the daily expense, mostly could maintain daily living
    □Balanced
    □More than balanced, has some deposit
    □Well-off
    □Affluent

II. Please describe your life experiences and feeling. (Please choose yes, no or not sure for following situations)

	Yes	No	Not sure
1. Have you ever stolen anything? (ex. stole money or things from others; stole things from stores)			
2. Did you ever run away from home overnight or once without returning for a lengthy period?			
3. Do you often tell lies or make up stories?			
4. Have you ever set up fire intentionally?			
5. Have you ever skip classes or truant from school before college?			
6. Have you ever broken into someone else’s house or car?			
7. Have you ever destroyed others’ property intentionally? (like break the window, scratch cars)			
8. Have you ever been physically cruel to animals?			
9. Have you ever forced someone into sexual activity or cause physical harms to someone in sexual activity?			
10. Have you ever initiate physical fights?			
11. Did you use weapons during fights? (ex. A bat, knife, iron bar)			
12. Did you ever trick or threaten others to obtain their properties?			
13. Have you ever hurt others intentionally? (ex. During fight or being physically cruel to others)			
14. Did you stay out at night despite parental prohibitions?			
15. Did you ever bully others? (including threaten, intimidate, torture, harassment, tease, bully others and cause suffering)			
16. Do you often loss your temper or could not control yourself to be mad at others and will shout, throw things or cause damage?			
17. Was there ever a time you would argue or talk back a lot with adults, your parents or teachers?			
18. Do you often deliberately defy or disobey adults’ requests or rules? (ex. Often refuse to do the things that your parents/teachers ask you to do)			
19. Often deliberately annoy others.			
20. When you did something wrong, you would often blame others for your misbehavior.			
21. People easily make you annoyed, or you are easily being offended by others.			
22. You are often angry or resentful.			
23. You have been spiteful or vindictive.			
24. When my friends invite me to KTV or karaoke, I will definitely go.			
25. I will definitely go to internet café with or without invitation.			
26. When there is house party, I will definitely go.			

	Yes	No	Not sure
27. When there is a chance to go to nightclub or pub, I will definitely go.			
28. When there is a chance to go play pool or video arcade, I will definitely go.			

III. Have your friends/classmates had the following conditions? (please choose a suitable item: all, most, half, few or none)

	**All=4**	**Most=3**	**Half =2**	**Few=1**	**None=0**
Study hard					
Follow the rules					
Habitual swearing					
Stealing, lying or extort					
Intimidate, extort, sexual assault or bully others					
Set on fire, engage in physical fight, abuse others or fight with weapons					
Destroy public property or infringe or destroy others' p property					
Being suspended from school					
Join gangs					
Being caught in the police station (except join student movement or car accident)					
Smoking					
Chew betel nuts					
Excessive drinking					
Ever use illicit substance					
Often use sedatives					
Often use prescription pain medicine					
Often use stimulant or refreshing drinks					
